# Risk of interstitial lung disease in patients treated for atrial fibrillation with dronedarone versus other antiarrhythmics

**DOI:** 10.1002/pds.5233

**Published:** 2021-05-04

**Authors:** Arlene Tave, Earl Goehring, Vibha Desai, Chuntao Wu, Rhonda L. Bohn, Sally G. Tamayo, Nicholas Sicignano, Juhaeri Juhaeri, Judith K. Jones, Sheila R. Weiss

**Affiliations:** ^1^ PharmaLex US Corp Fairfax Virginia USA; ^2^ HealthCore Andover Massachusetts USA; ^3^ Sanofi Pharmaceuticals, Inc. Bridgewater New Jersey USA; ^4^ Bohn Epidemiology Boston Massachusetts USA; ^5^ Naval Medical Center Portsmouth Virginia USA; ^6^ Health ResearchTx Trevose Pennsylvania USA; ^7^ Avigilan, LLC Potomac Maryland USA

**Keywords:** amiodarone, antiarrhythmia agents, atrial fibrillation, dronedarone, lung diseases, interstitial

## Abstract

**Purpose:**

To compare risks of interstitial lung disease (ILD) between patients treated with dronedarone versus other antiarrhythmics.

**Methods:**

Parallel retrospective cohort studies were conducted in the United States Department of Defense Military Health System database (DoD) and the HealthCore Integrated Research Database (HIRD). Study patients were treated for atrial fibrillation (AF) with dronedarone, amiodarone, sotalol, or flecainide. Propensity score matching was employed to create analysis cohorts balanced on baseline variables considered potential confounders of treatment decisions. The study period of July 20, 2008 through September 30, 2014 included a 1‐year baseline and minimum 6 months of follow‐up, for patients with drugs dispensed between July 20, 2009 and March 31, 2014. Suspect ILD outcomes were reviewed by independent adjudicators. Cox proportional hazards regression compared risk of confirmed ILD between dronedarone and each comparator cohort. A sensitivity analysis examined the effect of broadening the outcome definition.

**Results:**

A total 72 ILD cases (52 DoD; 20 HIRD) were confirmed among 27 892 patients. ILD risk was significantly higher among amiodarone than dronedarone initiators in DoD (HR = 2.5; 95% CI = 1.1–5.3, p = 0.02). No difference was detected in HIRD (HR = 1.0; 95% CI = 0.4–2.4). Corresponding risks in sotalol and flecainide exposure groups did not differ significantly from dronedarone in either database.

**Conclusions:**

ILD risk among AF patients initiated on dronedarone therapy was comparable to or lower than that of amiodarone initiators, and similar to that of new sotalol or flecainide users. This finding suggests that elevated ILD risk associated with amiodarone does not necessarily extend to dronedarone or other antiarrhythmic drugs.

**Key Points**

- Hospitalized ILD risk among AF patients initiated on dronedarone therapy was comparable to that of new sotalol or flecainide users.
- The known elevated ILD risk associated with amiodarone does not seem to extend to the entire AAD therapy class including dronedarone.

## 1 INTRODUCTION

Antiarrhythmic drugs are an important component in the treatment of atrial fibrillation (AF). They are often used in conjunction with anticoagulants, which are proven effective in decreasing the risk of thromboembolism and stroke in AF patients.^1^, ^2^ Dronedarone (Multaq®) received United States market approval in July 2009 for the treatment of AF and atrial flutter. Dronedarone is a benzofuran derivative with an electrophysiological profile resembling that of amiodarone, but with different relative effects on individual ion channels and with structural modifications intended to eliminate the non‐cardiovascular adverse effects of amiodarone.^3^, ^4^

Amiodarone is today one of the most common causes of drug‐induced interstitial lung disease (ILD) in registries, with a reported incidence of 1.2%–8.8%.^5^, ^6^, ^7^, ^8^, ^9^ In a retrospective study of 500 consecutive patients treated with amiodarone in Japan 40 patients (8%) presented signs of drug‐induced ILD during a mean follow‐up of 48 months.^10^ ILD has also been reported among patients using other antiarrhythmic agents, including cases identified in post‐marketing surveillance among persons using dronedarone.^11^ The purpose of this study was to determine if dronedarone is associated with an elevated risk of ILD, relative to other antiarrhythmic agents.

## 2 METHODS

The risk of ILD among patients using dronedarone was compared to the corresponding risk among patients using other antiarrhythmic agents by conducting two parallel epidemiological studies, the findings from both of which are reported here. These studies both utilized a retrospective cohort design where patient experience was recreated from existing healthcare and medical insurance records.

The studies were conducted in two distinct populations: (1) the United States Department of Defense Military Health System database (DoD), and (2) the HealthCore Integrated Research Database (HIRD). With the exception of variations to accommodate differences in the structure or representation of values in the two databases, the same methodology was employed in conduct of the two studies. An Epidemiology Steering Committee monitored study progress.

The study cohorts comprised adults diagnosed with AF with a new (first) prescription fill for one of four antiarrhythmic agents (dronedarone, amiodarone, sotalol, or flecainide) with a dispensing date between July 20, 2009 and March 31, 2014 (index prescription). Patients were required to have a 365 day period (baseline), defined by continuous eligibility in their health plan prior to and including the index prescription's first dispensing date (index date). An AF diagnosis was defined as having one or more medical services with an AF diagnosis code (ICD‐9‐CM diagnosis code 427.31) during the baseline period. Exclusion criteria were a diagnosis of cancer, organ transplant, HIV, ILD, pneumonia or sarcoidosis during the baseline period. Women who were or became pregnant in the 280 days immediately before or following the index date and patients with unknown gender were also excluded. To assure the index prescription was an incident prescription, patients were excluded if they were dispensed the same antiarrhythmic drug at any time during the baseline period. Patients with multiple study drug fills on the index date were also excluded. Use of non‐index study drugs during the baseline period was allowed.

The study outcome was a diagnosis of hospitalized ILD following the index date. Each patient's follow‐up period began the day after their index date and continued until the earliest of (1) first inpatient service with an ILD‐related diagnosis code, (2) death, (3) termination of health plan eligibility, or (4) the end of the study period. Any record of treatment in an acute care hospital or skilled nursing facility with a discharge diagnosis for any one of the ICD‐9‐CM codes 515, 516.3–516.37, 516.8 or 516.9^12^ was flagged for clinical review and adjudication.

Given the complexity of an ILD diagnosis, presence of these diagnostic codes was only used to screen for potential ILD. An independent panel of three established ILD expert clinicians individually reviewed the medical records of patients with flagged events in order to determine if there was a definitive clinical diagnosis of new onset (incident) ILD.^13^ Each clinician manually reviewed the de‐identified patient profiles, which consisted of patient demographics (i.e., age and gender) and chronological medical history (i.e., inpatient and outpatient encounters, diagnoses and procedures, and drug dispensings) for up to 1 year preceding the patient's initial study drug fill through the end of the patient's follow‐up period. Reviewers were blinded to specific study drug exposures (drug name, formulation and quantity dispensed), although the dispensing date and days supply were provided. In addition to relevant diagnoses, the panelists reviewed available information with regard to timing of the ILD event relative to a patient's ongoing medical and drug history. While laboratory and radiology test results were omitted from the profiles, as they were not available for all patients, ILD Adjudicators looked at the relative timing of orders for pulmonary function tests and pulmonary imaging as a proxy. The panelists independently assigned each event a determination of “Yes,” “No” or “Indeterminate” regarding its validity as a true ILD outcome. Unanimous decisions were considered final. Disagreements among the reviewers were adjudicated through a facilitated discussion with final status determined by majority vote. If the three panel members each reached a different conclusion after the discussion, then the event was assigned a final classification of “Indeterminate.” Only events confirmed as incident ILD were used in the analysis comparing the risks of ILD in patients treated with dronedarone versus other antiarrhythmics.

Propensity score matching (PSM) was used to control for unmeasured confounding in treatment decisions. Potentially confounding medical history and personal characteristics were identified from baseline in‐ and out‐patient records. Propensity scores (PS) were calculated from a logistic regression model using baseline covariates to predict the probability of being prescribed dronedarone versus a comparator drug. Variables included in that model were determined via stepwise processing, with variables entered through the step with the lowest Akaike Information Criterion value retained for the final PS model. The same covariates were employed in both databases to develop the reduced model from which the PS were calculated, although differences in the databases led to different variables being retained in the two reduced models. The reduced logistic regression models were derived from full models run against datasets combining all cohorts in the respective databases, and then independently fitted to three subsets respectively containing all the dronedarone patients and all patients from one of the comparator cohorts (see Table S1 for the list of covariates in the full and reduced PS models). PSM was conducted within each of the three sets of PS using a nearest‐neighbor‐within‐caliper matching algorithm. The caliper width was 0.2 times the standard deviation of the PS for each cohort matched to dronedarone, and matching was conducted without replacement. To ensure that each cohort would be compared to the same group of reference patients, a qualifying PS‐matched patient from each of the three comparator cohorts was required for a dronedarone patient to be included in the analysis. Dronedarone patients lacking a match in any one of the comparator cohorts were excluded. Therefore, the analytical (matched) datasets are smaller than, and not directly comparable to, the full sets of cohorts. Standardized differences were used to assess balance of baseline covariates between the dronedarone cohort and each of the comparator cohorts in the PS matched datasets.

The risk of ILD was modeled separately for dronedarone versus each of the three comparators using Cox Proportional Hazards (CPH) regression. Separate CPH models used exposure group (dronedarone as the reference drug) as the main independent variable predicting risk of a confirmed ILD outcome. The models were adjusted for confounders that remained unbalanced between the dronedarone and respective comparator cohorts after PSM.

Recognizing the non‐specific clinical presentations of ILD, which may prevent a definitive diagnosis (e.g., cough, non‐specific pathology upon chest X‐ray), a sensitivity analysis was conducted using a broader definition of suspected ILD (i.e., addition of ICD‐9‐CM Codes 495.9 and 518.82). The sensitivity analysis was conducted only in the DoD cohorts and used the same methodology (i.e., PSM and CPH) as the primary analysis. The expanded definition of ILD was also applied to the baseline eligibility criteria, which resulted in 9580 patients being reclassified as prevalent for ILD and excluded from the analysis.

## 3 RESULTS

In the DoD database there were 37 704 eligible patients; the majority had amiodarone as their Index Drug (n = 18 521, 49.1%), followed by dronedarone (n = 8128, 21.6%), sotalol (n = 6140, 16.3%), and flecainide (n = 4913, 13.0%). Following PSM the final number in each matched cohort was 4087 (Table 1).

**TABLE 1:** Baseline characteristics of dronedarone patients in the ILD propensity score matched analysis datasets: DOD database—July 20, 2009 to September 30, 2014

Characteristics, N %	Dronedarone		Amiodarone		Sotalol		Flecainide	
	N	%	N	%	N	%	N	%
All patients passing screening	8128		18 521		6140		4915	
All PS‐matched patients	4087	*100*	4087	*100*	4087	*100*	4087	*100*
Demographics								
Gender (N, % female)	1994	*48.8*	1982	*48.5*	1940	*47.5*	2018	*49.4*
Age								
18–39 years	54	*1.3*	48	*1.2*	47	*1.1*	108	*2.6*
40–49 years	187	*4.6*	116	*2.8*	104	*2.5*	228	*5.6*
50–59 years	560	*13.7*	436	*10.7*	355	*8.7*	548	*13.4*
60–69 years	1478	*36.2*	1297	*31.7*	1293	*31.6*	1436	*35.1*
70–79 years	1355	*33.2*	1447	*35.4*	1540	*37.7*	1281	*31.3*
80+ years	453	*11.1*	743	*18.2*	748	*18.3*	486	*11.9*
History of disease								
Deyo‐Charlson Index, Mean (SD)	1.1	*1.5*	1.6	*1.7*	1.4	*1.6*	0.9	*1.3*
Asthma diagnosis	385	*9.4*	401	*9.8*	395	*9.7*	420	*10.3*
Bronchitis diagnosis	311	*7.6*	387	*9.5*	397	*9.7*	311	*7.6*
COPD diagnosis	527	*12.9*	704	*17.2*	638	*15.6*	415	*10.2*
Connective tissue disease diagnosis	149	*3.6*	157	*3.8*	148	*3.6*	171	*4.2*
Exposure to occupational or environmental toxins diagnosis	11	*0.3*	5	*0.12*	7	*0.2*	13	*0.3*
GERD diagnosis	1115	*27.3*	1149	*28.1*	1169	*28.6*	1128	*27.6*
History of medication use								
Other antiarrhythmic agents	20	*0.5*	22	*0.5*	19	*0.5*	15	*0.4*
Agents associated with drug‐induced pulmonary disease	3676	*89.9*	3792	*92.8*	3761	*92.0*	3682	*90.1*
Therapeutic oxygen treatment	0	*0*	1	*0.02*	1	*0.02*	0	*0*

Applying the same inclusion and exclusion criteria to the HIRD data resulted in a total of 26 165 eligible patients. Like the DoD database, the largest index drug cohort was amiodarone (n = 12 615, 48.2%; Table 2). The remaining cohorts each had similar numbers of eligible patients; 4894 (18.7%), 4556 (17.4%), and 4100 (15.7%) for sotalol, flecainide and dronedarone, respectively. Upon PSM, each Index Drug cohort contained 2886 patients.

**TABLE 2:** Baseline characteristics of dronedarone patients in the ILD propensity score matched analysis datasets: HIRD database—July 20, 2009 to September 30, 2014

	Dronedarone		Amiodarone		Sotalol		Flecainide	
Characteristics, N %	N	%	N	%	N	%	N	%
All patients passing screening	4100		12 615		4894		4556	
All PS‐matched patients	2886	*100*	2886	*100*	2886	*100*	2886	*100*
Demographics								
Gender (N, % female)	966	*33.5*	813	*28.2*	1078	*37.4*	1247	*43.2*
Age								
18–39 years	48	*1.7*	72	*2.5*	78	*2.7*	86	*3.0*
40–49 years	257	*8.9*	252	*8.7*	225	*7.8*	284	*9.8*
50–59 years	858	*29.7*	759	*26.3*	777	*26.9*	839	*29.1*
60–69 years	991	*34.3*	938	*32.5*	937	*32.5*	1002	*34.7*
70–79 years	502	*17.4*	583	*20.2*	605	*21.0*	486	*16.8*
80+ years	230	*8.0*	282	*9.8*	264	*9.2*	189	*6.6*
History of disease								
Deyo‐Charlson Index, Mean (SD)	1.2	*1.4*	1.8	*1.7*	1.3	*1.5*	0.8	*1.1*
Asthma diagnosis	253	*8.8*	241	*8.4*	243	*8.4*	254	*8.8*
Bronchitis diagnosis	195	*6.8*	199	*6.9*	191	*6.6*	146	*5.1*
COPD diagnosis	270	*9.4*	287	*9.9*	280	*9.7*	183	*6.3*
Connective tissue disease diagnosis	93	*3.2*	69	*2.4*	88	*3.0*	83	*2.9*
Exposure to occupational or environmental toxins diagnosis	6	*0.2*	5	*0.2*	4	*0.09*	2	*0.05*
GERD diagnosis	524	*18.2*	468	*16.2*	537	*18.6*	506	*17.5*
History of medication use								
Other antiarrhythmic agents	14	*0.5*	14	*0.5*	12	*0.4*	12	*0.4*
Agents associated with drug‐induced pulmonary disease	2039	*70.7*	2250	*78.0*	2126	*73.7*	2084	*72.2*
Therapeutic oxygen treatment	568	*19.7*	544	*18.8*	534	*18.5*	507	*17.6*

Following PSM, the distributions of patient characteristics were generally similar across the drug cohorts in DoD, with a few exceptions. For example, elderly patients (80+ years of age) accounted for 18% of both the amiodarone and sotalol drug groups, compared to 11% of the dronedarone and flecainide groups. Also, 17.2% of patients in the amiodarone cohort had a history of chronic obstructive pulmonary disease (COPD) compared to 10.2% in the flecainide group, with sotalol (15.6%) and dronedarone (12.9%) falling between these rates. Standardized differences identified remaining imbalance after PSM for mean age on index date between the dronedarone and sotalol cohorts (67.3 years vs. 70.1 years, respectively), and for the 12‐month interval (beginning July 1, 2009) in which the patient index date fell between the dronedarone and amiodarone cohorts (2.9 years vs. 2.6 years, respectively). As described in the study methods, these unbalanced PS model variables were controlled for explicitly in the relevant CPH models. No explicit adjustments were made for the imbalances noted for age 80+ years or COPD, as those characteristics were not included as covariates in the reduced PS model.

The HIRD had less variation in the distribution of covariates following PSM compared to the DoD. Gender was an exception, with the proportion female ranging from a low of 28.2% to a high of 43.2%, in the amiodarone and flecainide drug groups, respectively. HIRD also had lower proportions female in all drug cohorts when compared to the DoD database. In DoD, the proportion female ranged between 47.5% and 49.4%. Despite this difference seen in Table 2, standardized difference calculations showed all PS model covariates adequately balanced between the HIRD cohorts after PSM.

There were 113 suspected ILD cases in DoD and 33 in HIRD, identified from electronic health records or diagnostic claims during the follow‐up period (Table 3). Adjudication resulted in a total of 72 confirmed ILD cases, 52 (46.0% of suspected) in DoD and 20 (60.6% of suspected) in HIRD.

**TABLE 3:** Suspected and confirmed cases of incident interstitial lung disease during follow‐up^a^: DoD database primary analysis, HIRD database, and DoD database sensitivity analysis—July 20, 2009 to September 30, 2014

	Dronedarone		Amiodarone		Sotalol		Flecainide>	
	N	%	N	%	N	%	N	%
DoD primary analysis (N = 4087/cohort)^b^								
Suspected hospitalized ILD	24	*100*	42	*100*	27	*100*	20	*100*
Confirmed cases^c^	9	*37.5*	23	*54.8*	10	*37.0*	10	*50.0*
HIRD Analysis (N = 2886/cohort)^d^								
Suspected hospitalized ILD	10	*100*	10	*100*	6	*100*	7	*100*
Confirmed cases^c^	7	*70.0*	6	*60.0*	3	*50.0*	4	*57.1*
DoD Sensitivity Analysis (N = 3594/cohort)								
Suspected hospitalized ILD	26	*100*	38	*100*	35	*100*	23	*100*
Confirmed cases^c^	8	*30.8*	16	*42.1*	11	*31.4*	10	*43.5*

^a^ The follow‐up period started the day after the Index Date and ended at the earliest occurrence of the ILD event, end of eligibility in the health plan, death or end of the study period.

^b^ Not shown in the table, 12 of the total 113 suspected ILD cases in the DoD were adjudicated as indeterminate.

^c^ Percent value is percent of suspected cases by cohort.

^d^ Not shown in the table, 5 of the total 33 suspected ILD cases in the HIRD were adjudicated as indeterminate.

**FIGURE 1:**
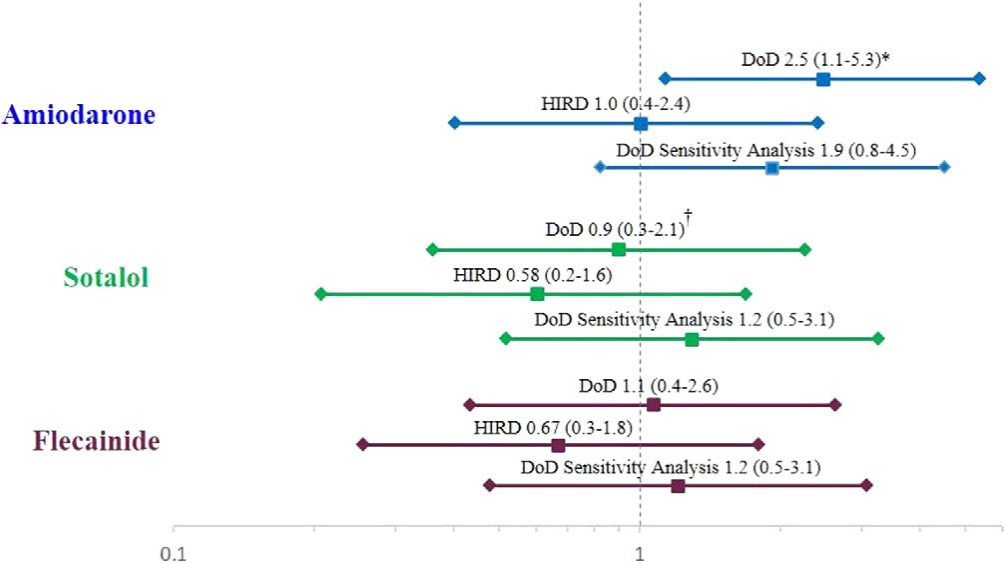
Hazard ratios and 95% confidence intervals of confirmed incident ILD in PS‐matched analysis datasets during follow‐up for new users of other antiarrhythmic medications relative to dronedarone: DoD database primary analysis, HIRD database, and DoD database sensitivity analysis—July 20, 2009 to September 30, 2014. * *P* < 0.05; Hazard ratio is adjusted for study year of index date. ^†^ Hazard ratio is adjusted for age on index date

As noted in the methodology, dronedarone was the reference group for all comparisons in the CPH models. In the DoD database, amiodarone exposure was associated with an elevated hazard of confirmed ILD (HR = 2.5; 95% CI = 1.1–5.3, *p* = 0.02) when compared to dronedarone exposure. None of the other comparison exposures differed significantly from dronedarone in terms of confirmed ILD risk in the DoD database. In the HIRD database there were no significant differences in risk of confirmed ILD between new users of any of the comparator therapies versus dronedarone. Contrary to the significantly higher risk of confirmed ILD associated with amiodarone than dronedarone in the DoD database, in the HIRD database the HR for amiodarone was 1.0 (95% CI = 0.4–2.4; Figure 1).

The sensitivity analysis in the DoD cohorts flagged 122 potential ILD cases, of which 45 (36.9%) were confirmed upon adjudication. Because the sensitivity analysis used an expanded list of codes to screen for potential ILD events, and that list was applied to the entire observation period, the presence of one of these codes in the baseline period resulted in the exclusion of some patients who were classified as ILD cases in the primary analysis. CPH analyses identified no differences in the risk of confirmed ILD between the dronedarone cohort and any of the three comparator therapy cohorts.

## 4 DISCUSSION

This retrospective cohort study found the hazard of confirmed ILD among AF patients initiated on amiodarone therapy to be 2.5 times that of new dronedarone users in the larger DoD, and comparable between those cohorts in HIRD. No increased ILD risk was observed between new users of dronedarone versus sotalol or flecainide in either database. These results are in concert with the drug's design, and consistent with previous population‐based post‐marketing analyses of dronedarone.^14^

A key strength of this study was the ability to thoroughly identify pre‐ and post‐exposure events in two large longitudinal databases, which combined with PSM to create analysis cohorts balanced on baseline risk factors. A sensitivity analysis conducted in the larger DoD database reinforced the primary findings.

This study is distinguished from previous research by its analysis of only events confirmed as ILD by expert clinicians. Adjudication of suspected cases was a critical element as ILD is not clearly identified in EHR and claims databases. Fewer than half of suspected ILD cases in the DoD and less than two‐thirds in HIRD were confirmed. While more suspected cases were flagged in a sensitivity analysis using a broader ILD definition, fewer total cases were confirmed in that exercise as some suspected incident cases from the primary analysis were screened out as prevalent at baseline under the expanded definition. Adjudicators evaluated all suspected cases based on their considerable diagnostic experience, although precision of the assessments would have benefitted from availability of relevant test results (e.g., pulmonary function tests and/or lung scan results) and more complete information for conditions where data were limited or possibly underreported (e.g., smoking).

Comparing the risk of ILD across treatments, particularly in database studies, is hampered by the complexity of a clinical diagnosis and low specificity of diagnostic codes, which limits analysis to databases with access to medical records. This limitation was evidenced by the paradoxically decreased number of confirmed ILD cases in the sensitivity analysis, where expanding the definition of ILD to include non‐specific or otherwise unclassified pulmonary conditions resulted in reclassification of some previously presumed incident cases. These analyses intentionally focused on patients with disease severe enough to require inpatient care, utilizing diagnostic codes to screen patient records for potential new diagnoses of ILD which were then confirmed through adjudication by clinical experts.

Dronedarone was approved with a Risk Evaluation and Mitigation Strategy (REMS) that includes periodic assessment of education efforts regarding the drug's risks.^15^ Although the effect of the REMS on clinical practice could not be explored in this study, low incidence of ILD in these two large populations might reflect care taken to monitor potential disease symptoms and pulmonary function, and discontinue treatment in patients with concerning findings before their conditions worsened. Low outcome rates are also consistent with the relatively small proportions of patients with baseline risk factors, which might also suggest careful attention to prescribing information. In addition, the similarly low prevalence of baseline risk factors across the comparator cohorts might suggest that the REMS accompanying dronedarone's approval could have affected prescribing patterns for these other antiarrhythmic drugs.

In conclusion, dronedarone was associated with comparable or significantly lower risk of hospitalized ILD than amiodarone therapy in AF patients, and with consistently comparable hospitalized ILD risk in similar patients initiated on sotalol or flecainide. The data suggest that the elevated risk of hospitalized ILD associated with amiodarone use in AF patients does not necessarily extend to dronedarone and other antiarrhythmic drugs.

## CONFLICT OF INTEREST

Ms. Tave, Mr. Goehring, Dr. Desai, Dr. Bohn, Dr. Jones and Dr. Weiss declare that they have no conflicts of interest. Dr. Wu was employed by Sanofi at the time of conducting this study. Dr. Juhaeri is a Sanofi employee. Dr. Tamayo is a retired member of the US military; this work was prepared as part of her official duties. Mr. Sicignano is an employee of Health ResearchTx.

## ETHICS STATEMENT

This study did not involve human participants.

## DISCLAIMER

Research data derived from an approved Naval Medical Center, Portsmouth, Virginia IRB, protocol; number NMCP.2014.0044. The views expressed in this manuscript reflect the results of research conducted by the authors and do not necessarily reflect the official policy or position of the Department of the Navy, Department of Defense, or the United States Government. Copyright Notice: CAPT Sally Tamayo, MC, USN, (Ret.) was a military service member. This work was prepared as part of her official duties. Title 17 U.S.C. 105 provides that “Copyright protection under this title is not available for any work of the United States Government.” Title 17 U.S.C. 101 defines a United States Government work as a work prepared by a military service member or employee of the United States Government as part of that person's official duties.

## Supporting information

**Supplemental Table S1** Baseline Characteristics Included in Full and Reduced Propensity Score Models
