# Supplementary material for: Risk of interstitial lung disease in patients treated for atrial fibrillation with dronedarone versus other antiarrhythmics
Source: Pharmacoepidemiol Drug Saf. 2021 May 4;30(10):1353–9. doi: 10.1002/pds.5233 (PMC8453764; doi:10.1002/pds.5233)
Supplement: Supplementary file 1 — Supplemental Table S1 Baseline Characteristics Included in Full and Reduced Propensity Score Models [file PDS-30-1353-s001.docx]

**Supplemental Table 1. Baseline Characteristics Included in Full and Reduced Propensity
Score Models**

|  | **Included in reduced models** | | |
| --- | --- | --- | --- |
| **Characteristics in full logistic regression model** | **DoD** | **HIRD** | **DoD Sensitivity Analysis** |
| Age (continuous) | **X** | **X** | **X** |
| Gender (1=male) | **X** | **X** | **X** |
| Study year (12-month period from 7/1/2009 where Index Date falls [1,2,3,4 or 5]) | **X** | **X** | **X** |
| Any Hospitalization | **X** | **X** | **X** |
| Any Emergency Room Visit | **X** |  |  |
| Number of office visits (grouped by quartile) | **X** | **X** | **X** |
| Pulmonologist Visit | **X** | **X** |  |
| Number of other non-study medications |  |  |  |
| Number of Different Drugs Dispensed in Baseline by Therapeutic Class –Grouped by Quartile | **X** | **X** | **X** |
| ***History of condition, procedure or medication therapy:*** | | | |
| Asthma | **X** | **X** | **X** |
| Bronchitis |  |  |  |
| Chronic Obstructive Pulmonary Disease |  |  |  |
| Connective Tissue Disease |  |  |  |
| Any toxin exposure |  |  |  |
| Gastroesophageal Reflux Disease | **X** |  | **X** |
| Therapeutic Oxygen Treatment | **X** | **X** | **X** |
| One Or More Study Drugs Dispensed | **X** | **X** | **X** |
| Other Antiarrhythmic Therapy Dispensed |  |  |  |
| Potentially Interstitial Lung Disease Inducing Drug Dispensed | **X** | **X** | **X** |
